# Sand dust image visibility enhancement algorithm via fusion strategy

**DOI:** 10.1038/s41598-022-17530-3

**Published:** 2022-08-02

**Authors:** Yazhong Si, Fan Yang, Zhao Liu

**Affiliations:** School of Electronic and Information Engineering, Hebei University of Technology, Tianjin, 300401 China

**Keywords:** Electrical and electronic engineering, Imaging and sensing

## Abstract

The outdoor images captured in sand dust weather often suffer from poor contrast and color distortion, which seriously interfere with the performance of intelligent information processing systems. To solve the issues, a novel enhancement algorithm based on fusion strategy is proposed in this paper. It includes two components in sequence: sand removal via the improved Gaussian model-based color correction algorithm and dust elimination using the residual-based convolutional neural network (CNN). Theoretical analysis and experimental results show that compared with the prior sand dust image enhancement methods, the proposed fusion strategy can effectively correct the overall yellowing hue and remove the dust haze disturbance, which provides a constructive idea for the future development of sand dust image enhancement.

## Introduction

In sandstorm weather, the images captured by imaging equipment always have such issues as color distortion, low contrast, and low recognizability. Affected by Mie scattering, the blue–violet light is absorbed by sand dust particles much more quickly compared with red and orange light, which plays an adverse role in various remote-based computer vision tasks and seriously interferes with the performance of intelligent information processing systems. Therefore, the image quality enhancement technology for sand dust images is an important research topic in the field of image processing and computer vision.

At present, fusion algorithms have a wide range of applications in image processing^1–4^. However, such a fusion strategy on image enhancement in bad weather mainly focuses on image dehazing^5–8^, image deraining^9,10^, and image desnowing^11,12^. To our best knowledge, there is no well-known reported work related to sand dust removal for outdoor scene images up till now, which highlights the value of our research. The current image sand dust removal algorithms can be roughly divided into image restoration^13–17^ and image enhancement^18–22^ two mainstream categories.

Image restoration methods mainly rely on the atmospheric scattering model^24^. These algorithms employ the prior knowledge^25^ to estimate the intermediate parameters, which are then substituted into the atmospheric scattering model to recover the clear images. Image enhancement algorithms improve the clarity of sand dust degraded images via the prior image processing theory. Such as balancing the histogram equalization^19,21^, guided filtering^22,26^, and Retinex-based filtering^18,27^.

There are still some issues that cannot be ignored. For the physical model-based methods, inaccurate intermediate parameters may lead to dark tones and artifacts in the output images. For image enhancement algorithms, these methods are only suitable for a certain sandstorm scene. Many sand dust enhancement algorithms will fail if the distribution of sand dust images is too complex.

Recent years have witnessed the great success of CNN, which has attracted widespread attention from the field of image processing. Numerous researchers designed the enhancement algorithms based on CNN to handle the degraded images in complex environments^5–12^, which all benefited from the public datasets^28–32^. However, the absence of available sandstorm datasets hinders the development of CNN-based sand dust image enhancement algorithms.

**Figure 1:**
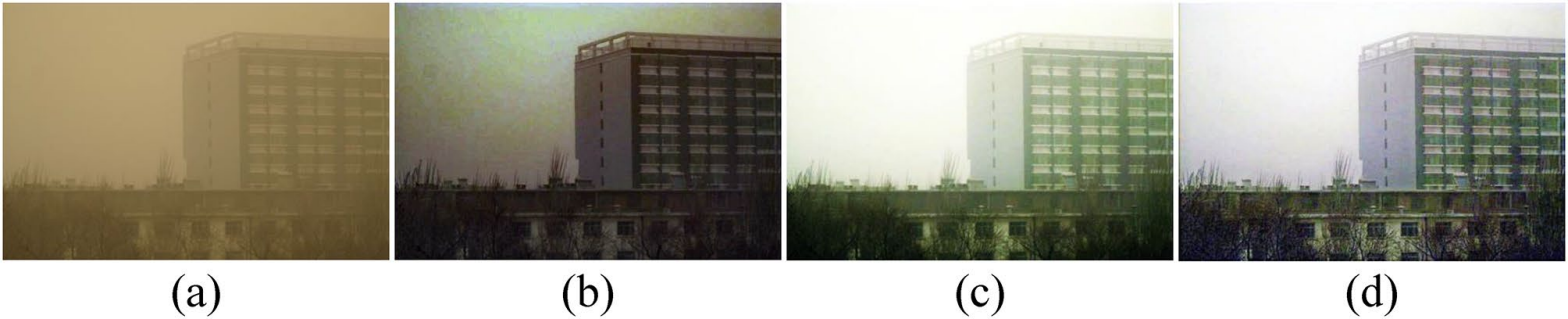
The comparison of real-world sand dust removal. (**a**) Sand dust image; (**b**) CIDC^16^; (**c**) TTFIO^23^; (**d**) The proposed method.

To bridge the gaps, we proposed a fusion strategy that includes both color correction and image reconstruction. The improved color correction algorithm based on the Gaussian model is used to remove the visual distraction of sand. We then transform the dust removal task into a haze removal task to further improve the clarity of the input images. The CNN based on the residual structure is designed to eliminate the dust nonlinearly, and we provide a comparison result on the real-world sand dust image, as shown in Fig. 1.

The main contributions of this work are summarized as follows:

- We proposed a simple but effective fusion strategy for combining the conventional image processing algorithm with CNN to improve the clarity of the sand dust images. The proposed fusion algorithm provides a novel way for future research on sand dust image enhancement.
- A color correction algorithm based on the Gaussian model is improved in our method to balance the hue of degraded sand dust images.
- We designed a novel CNN built on an autoencoder-like structure, which can effectively improve the local area brightness and reduce the loss of detail information in the training process.

The remainder of this paper is organized as follows. The existing sand dust removal algorithms are introduced in “Related work” section. The details of the proposed sand dust removal method are described in “Proposed method” section. The experimental setup implemented details and evaluation results of the proposed method are illustrated in “Experimental results and analysis” section. And we will further analyze and discuss the details of the proposed fusion algorithm in “Ablation study” section. Finally, the conclusion is presented in “Conclusion” section.

## Related work

### Sand dust image restoration algorithms

There are two categories in current sand dust removal algorithms, including image restoration and image enhancement. The sand dust image restoration algorithms were designed based on the atmospheric scattering model, which is widely used to describe the imaging process in haze weather. Mathematically, the structure of the physical model can be expressed as:

1 $$\begin{aligned} I(x) = J(x)t(x) + A(1 - t(x)) \end{aligned}$$

where *I*(*x*) is haze image; *J*(*x*) denotes corresponding haze-free image; *A* is the global atmosphere light of the haze image; *t*(*x*) is the transmission. The sand dust image can be treated as a haze image with color distortion. Therefore, we described the degradation model as the following:

2 $$\begin{aligned} {I{_s}(x)} = D\left[ {J(x)t(x) + A(1 - t(x))} \right] \end{aligned}$$

where $I_s(x)$ is the degraded sand dust image; *D*(*x*) is the color degradation model. To restore a clear image, the sand dust image degradation model can be transformed into the following:

3 $$\begin{aligned} J(x) = A + \frac{{{D^{ - 1}}\left[ {I{_s}(x)} \right] - A}}{{t(x)}} \end{aligned}$$

In image restoration algorithms, researchers designed color correction algorithms to balance the hue of degraded sand dust images. Then, they estimated the necessary parameters via DCP^25^. Finally, they substituted the parameters into the degradation model to restore the dust-free images. Dhara et al.^16^ proposed a dehazing algorithm using weighted least squares filtering on dark channel prior and color correction that involves automatic detection of color cast images. Peng et al.^14^ proposed a new approach to estimating ambient light which is needed by the DCP restoration methods and added adaptive color correction into the degradation model to stretch the contrast of the image while solving the issue of color distortion. Considering the attenuation of the blue channel may cause the DCP method to fail, Gao et al.^17^ proposed a sand dust image restoration method based on reversing the blue channel prior (RBCP). Shi et al.^15^ proposed an algorithm based on halo-reduced DCP dehazing for sand dust image enhancement, they corrected color in the LAB color space based on gray world theory, removed the dust using a halo-reduced DCP dehazing method, and contrasted stretching in the LAB color space using a Gamma function.

### Sand dust image enhancement algorithms

The other mainstream method is the image enhancement algorithm. Many researchers enhance the sand dust image quality by balancing the histogram and adjusting the contrast of the degraded image. Fu et al.^18^ proposed a retinex-based enhancing approach, they adopted an effective alternating direction optimization strategy to solve the proposed model. Cheng et al.^22^ compensated the loss value in the blue channel, and enhanced the image contrast and edge information through guided image filtering. Shi et al.^19^ proposed a normalized gamma transformation-based contrast-limited adaptive histogram equalization (CLAHE) with color correction in Lab color space for sand-dust image enhancement. Wang et al.^21^ proposed a fast color balance method followed by a fusion model to enhance the sandstorm-degraded images.

Sand dust images have narrow histogram distribution. The current enhancement methods adopted image processing theory to balance the color and contrast of the images can only improve the clarity of the images to a certain extent, and the balanced images are still hazy in vision. Moreover, the manually designed prior knowledge based on specific observations may not always suit the inherent properties of the degraded sandstorm images.

## Proposed method

**Figure 2:**
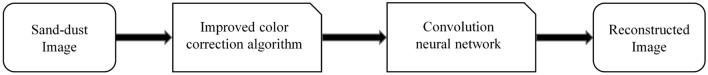
The framework of the fusion strategy. The proposed method combines conventional algorithms with deep learning to further improve the clarity of sand dust images.

**Figure 3:**
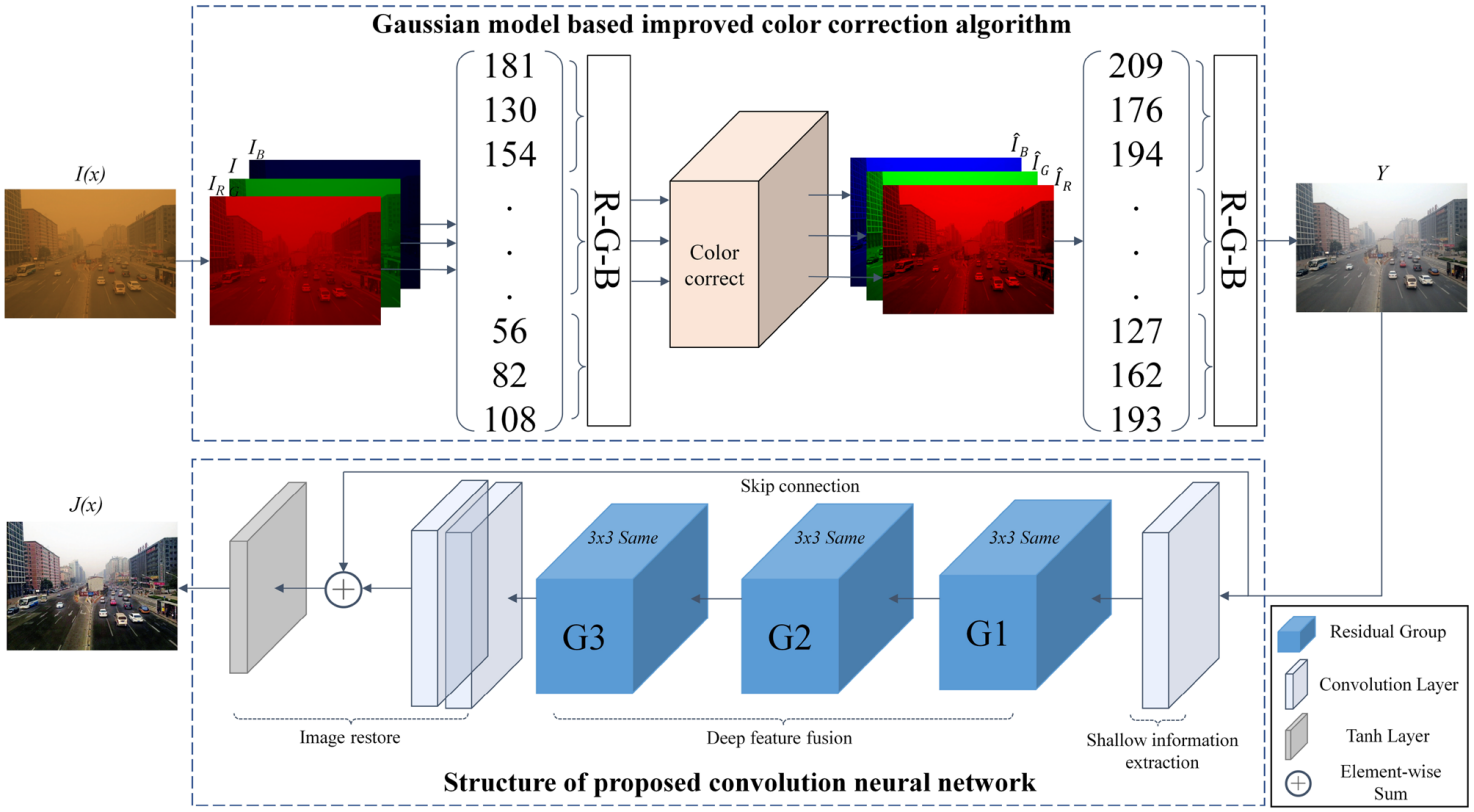
The architecture of the proposed sand dust removal algorithm.

Currently, the mainstream methods of sand dust removal are based on traditional algorithms, most of them may corrupt the image textures and over smooth the detail information. To the best of our knowledge, there is no excellent reported fusion method related to sand dust removal for outdoor scene images. In view of the shortcomings of the conventional sand dust removal algorithms, we proposed a novel fusion algorithm to combine color correction algorithm with deep learning, which can further improve the visibility of sand dust degraded images. The framework of the proposed method is shown in Fig. 2, and the schematic illustration is shown in Fig. 3. The proposed algorithm includes a color correction pre-processing algorithm and a learning-based dust elimination network. The skip connection in the network can improve the robustness and accelerate the convergence speed of the network. Experiments demonstrate that the results of the proposed method have richer detailed information and are closer to the reference images.

### Sand removal via Gaussian model-based improved color correction algorithm

Experimental results show that direct processing of degraded sand dust images using CNN does not yield satisfactory results. There is still a certain degree of color deviation in the output, and more detail will be discussed in the ablation study.

Therefore, it is still necessary to preprocess the sand dust degraded images. According to the prior characteristics of the sand dust image, Zhi et al.^33^ proposed an image color correction algorithm based on the Gaussian model. The algorithm only uses the G component as the benchmark to calculate the extension coefficient. However, there are some disadvantages in the strategy of using only a single channel distribution as a reference. The corrected results are susceptible to the G component, if the G component with wide or polarization histogram distribution, the original method will suppress the contrast of the image, which may cause the pixel values to overflow or underflow, resulting in local information being lost and color degraded.

**Figure 4:**
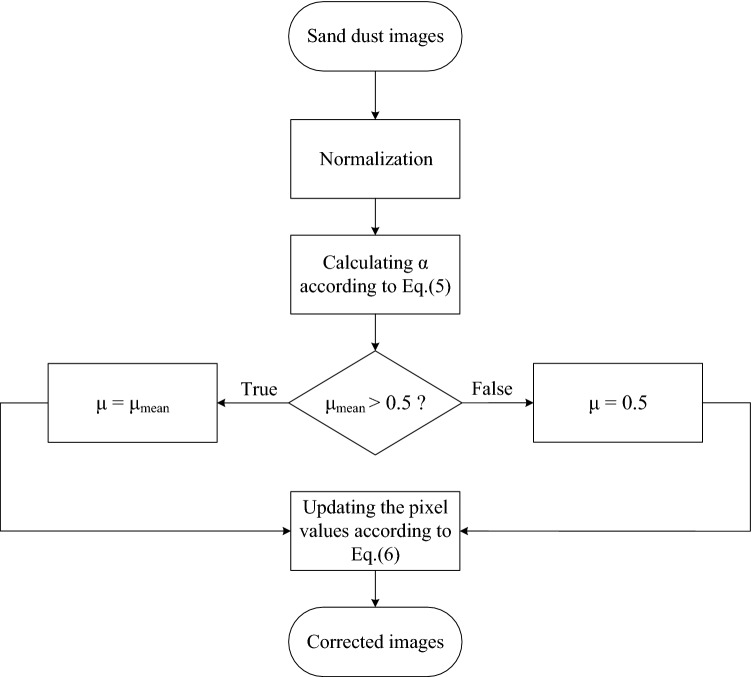
The flow chart of the Gaussian model-based improved color correction algorithm, please refer to the text for more detailed steps.

To solve the above-mentioned issues, we reprogrammed the adaptive color adjustment strategy with the pixel difference in each channel of RGB as a reference to adjust the pixel values for each channel separately. Meanwhile, for the characteristics of low luminance and low contrast of sand dust images, we enhanced the images whose luminance mean value is less than the reference luminance to highlight the original local details of the images. The flow chart of the Gaussian model-based improved color correction algorithm is shown in Fig. 4, and more specific steps as the following:

*Step 1* Normalizing R, G, B components pixel values of input $I_C$, then calculating the average value $\mu _c$ and the average standard deviation $\sigma _c$ of each channel respectively. The normalization process is as follows:

4 $$\begin{aligned} {\hat{I}}_C = \frac{{({I_C} - \min {I_C})}}{{\left( {\max {I_C} - \min {I_C}} \right) }},C \in \left\{ {R,G,B} \right\} \end{aligned}$$

*Step 2* Calculating the extension coefficient $\alpha$ which is defined as:

5 $$\begin{aligned} \alpha = \frac{1}{{{{\max }_C}\left( {{\hat{I}}_{\max }^{_C} - {\hat{I}}_{\min }^{_C}} \right) }},C \in \left\{ {R,G,B} \right\} \end{aligned}$$

where ${\hat{I}}_{max}^c$ and ${\hat{I}}^c_{min}$ are the maximum and minimum pixel values in channel *C*, respectively. We adopted the maximum value of pixel difference in R, G, and B components as a reference to calculate $\alpha$.

*Step 3* Updating RGB channels pixel values of the color-biased images by the following:

6 $$\begin{aligned} {I'_C} = \mu + \alpha \times \frac{\sigma }{{{\sigma _C}}} \times \left( {{{\hat{I}}_C} - {\mu _C}} \right) \end{aligned}$$

7 $$\begin{aligned}&\sigma \mathrm{{ = }}\sum \limits _{C \in \left\{ {R,G,B} \right\} } {\frac{{{\sigma _C}}}{3}} \end{aligned}$$

8 $$\begin{aligned}&\mu = \left\{ \begin{array}{l} 0.5{} {} {} {} {} {} {} {} {} {} {} ,{} {} {} {} {} {} {} {} {} {} {\mu _{\mathrm{{mean}}}}{} {} \le 0.5\\ {} {\mu _{\mathrm{{mean}}}},{} {} {} {} {} {} {} {} {} {} {} {} {\mu _{\mathrm{{mean}}}} > 0.5 \end{array} \right. \end{aligned}$$

9 $$\begin{aligned}&{\mu _{\mathrm{{mean}}}}\mathrm{{ = }}\sum \limits _{C \in \left\{ {R,G,B} \right\} } {\frac{{{\mu _C}}}{3}} \end{aligned}$$

where $\mu$ is the brightness reference coefficient, it can fix the color position center of RGB to avoid the issues of contrast reduction and image information loss caused by pixel overflow or underflow; $\sigma$ and $\mu _{mean}$ are the average standard deviation and the mean value of color-biased image respectively, which can describe the relative concentration degree of RGB color values and evaluate the image quality to a certain extent. Equation (8) can enhance the detail of dark images while preserving the luminance information of bright images.

Assuming *I* is the sand dust image; *G* is the color correction model; *Y* is the output of *G*. The improved color deviation correction algorithm based on the Gaussian model can be simplified as:

10 $$\begin{aligned} Y\mathrm{{ = }}G\mathrm{{(}}I\mathrm{{)}} \end{aligned}$$

**Figure 5:**
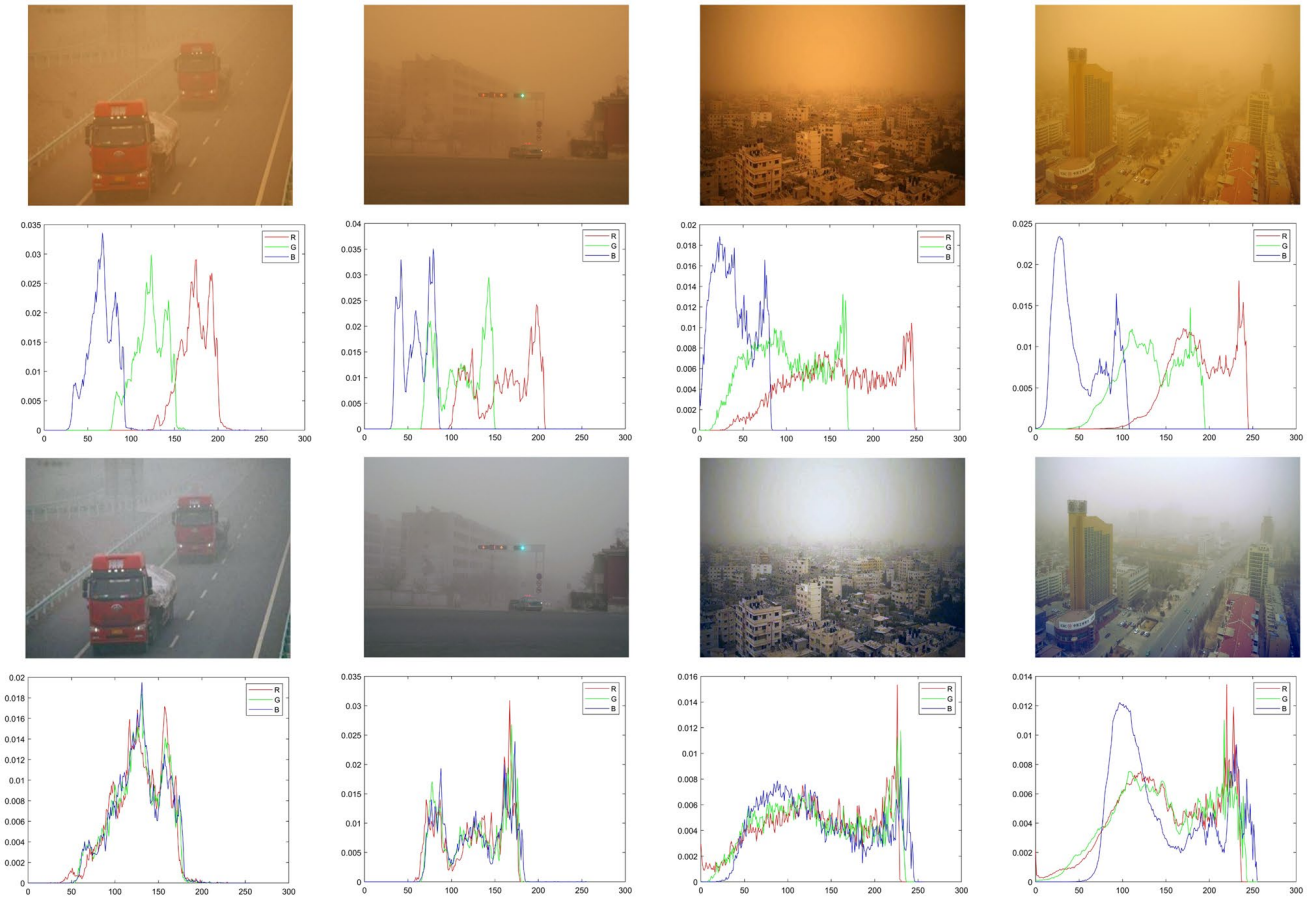
The comparisons of histogram distribution before and after color shifting correction of real-world sand dust image, the issue of color distortion is effectively solved and the histogram distribution of RGB channels is balanced.

The histogram distribution of sand dust images has obvious prior characteristics for shifting, concentration and sequence. The shifting refers to that the histogram distribution of RGB channels is dispersed. Concentration means that the pixel values in RGB channels are concentrated in a certain gray range respectively. Sequence refers to the histogram distribution of RGB channels in order according to B, G, R. Figure 5 shows the results of the improved color correction algorithm. The top two rows show the sand dust images and their corresponding histogram distributions, and the bottom two rows present the images and their corresponding histograms after processing by the improved algorithm. In Fig. 5, the images processed by the improved algorithm are better corrected both in vision and mathematical statistics.

### Image dust removal based on CNN

After being processed by the color correction algorithm, the distribution of the outputs is similar to the hazy images. Thus, to further improve the image visibility and the universality of the algorithm, we designed a simple yet effective end-to-end network.

#### The architecture of the proposed network

The unit in the network includes a convolution layer, a normalization layer, and an activation layer. The skip connections in the model can retain more information flow in the training process. The residual block in the deep feature fusion module can effectively solve the issue of gradient diffusion and improve the robustness of the network.

The schematic illustration of the proposed network is shown at the bottom of Fig. 3. The network is composed of three parts: shallow information extraction module, deep feature fusion module, and image reconstruction module. The shallow information extraction module preliminarily extracts the low-dimensional information of the input. The deep feature fusion module is composed of *N* residual groups, each group contains *m* residual blocks for the deep features fusion of the image. Assuming *n* is the dimension of the feature vector and $R^n$ is the set of n-dimensional space. The n-dimensional feature vector in the process of deep feature fusion can be expressed as:

11 $$\begin{aligned} x = {\left( {{v_1},{v_2},{v_3}, \ldots ,{v_n}} \right) ^T},x \in {R^n} \end{aligned}$$

**Figure 6:**
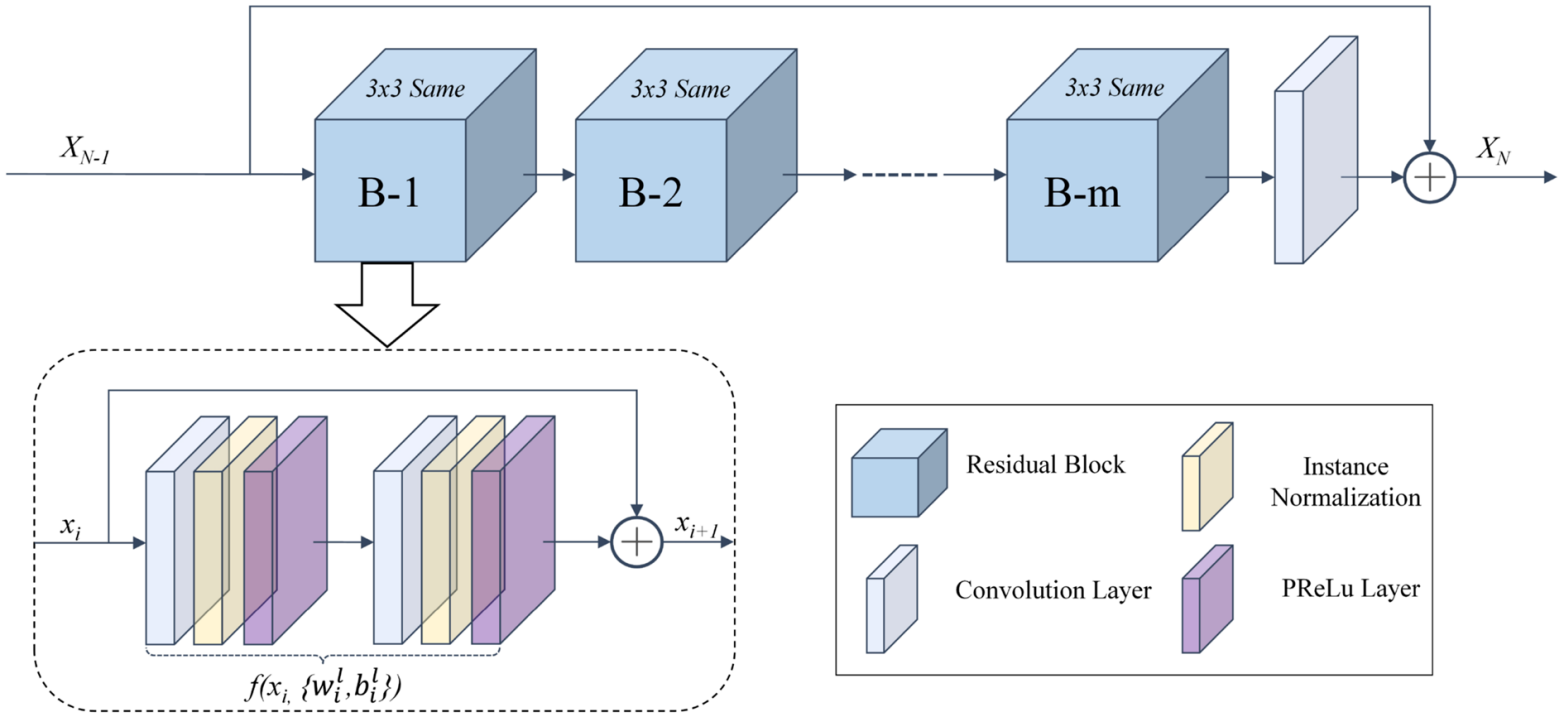
Structure of group residual, the skip connection can effectively preserve the local details and original features.

The residual group structure is shown in Fig. 6. We described the residual block as the following:

12 $$\begin{aligned}&{x_{i + 1}} = {x_i} + f\left( {x_i},\left\{ w_i^l,b_i^l\right\} \right) \end{aligned}$$

13 $$\begin{aligned}&f\left( {x_i},\left\{ w_i^l,b_i^l\right\} \right) = w_i^2\delta \left( r\left( w_i^1{x_i} + b_i^1\right) \right) + b_i^2 \end{aligned}$$

where $x_i$ is the input of the *i*th residual block and the output is $x_{i+1}$. *f*(*x*) represents the output of the residual block; $w_i^l$ and $b_i^l$ are the weight parameter and bias of layer *l* in *i*th block respectively; $\sigma$ is PReLu activation layer and *r* represents the instance normalization layer. The deep feature fusion module is composed of *N* residual groups, and each residual group contains *m* residual blocks. Therefore, the output $X_N$ of the *N*th residual group can be deduced from Eq. (13) as follows:

14 $$\begin{aligned} {X_N} = {X_{N - 1}} + \sum \limits _{i = 1}^m {{f_N}\left( {x_i},\left\{ w_i^l,b_i^l\right\} \right) } \end{aligned}$$

The image reconstruction module integrates the deep features with the shallow information, which can improve the network’s generalization capabilities. The experimental results show that the proposed network can effectively solve the gradient dispersion issue and further improve the clarity of the color-corrected images.

#### Loss function

In this paper, we designed a novel objective function including L1-regularized and structural similarity (SSIM) loss. L1 loss is widely used to minimize the difference between the output and the reference image, which can be written as:

15 $$\begin{aligned} {L_1} = \frac{1}{n}\sum \limits _{i = 1}^n {\left\| {{J_i} - {X_i}} \right\| } \end{aligned}$$

where *J* is the reconstructed image; *X* is the reference image and *n* is the number of training data.

SSIM is commonly used to evaluate the similarity of two images. In our network, we took SSIM loss as a sub-constrain, which can be written as follows:

16 $$\begin{aligned}&{L_{SSIM}} = 1 - SSIM\left[ {J,X} \right] \end{aligned}$$

17 $$\begin{aligned}&SSIM\left[ {J,X} \right] = \frac{{2{\mu _J}{\mu _X} + {C_1}}}{{\mu _{_J}^2 + \mu _X^2 + {C_1}}} \cdot \frac{{2{\sigma _{JX}} + {C_2}}}{{\sigma _J^2 + \sigma _X^2 + {C_2}}} \end{aligned}$$

where $\mu _x$ and $\sigma _x^2$ represent the mean and variance of *x* respectively; $\sigma _{xy}$ is the covariance between *x* and *y*; $C_1$ and $C_2$ are the constants to keep the calculation stable.

We integrated the above loss functions and assumed that $\lambda _1$ and $\lambda _2$ are the corresponding trade-off weights. The expression of the total loss function is as follows:

18 $$\begin{aligned} {L_{\mathrm{{total}}}} = {\lambda _1}{L_1} + {\lambda _2}{L_{SSIM}} \end{aligned}$$

## Experimental results and analysis

### Training and testing datasets

Datasets can affect network performance to a great extent. However, it is difficult to collect a large number of paired sand dust samples in reality for training the network. At present, the mainstream dehazing methods based on deep learning train the network through synthetic haze images. The common dehazing dataset including NYU-Depth^28^, NH-Haze^29^ and RESIDE^30^.

In this paper, the real-world sand dust images in the experiments all come from the dataset constructed by Shi et al.^15^. Moreover, we adopted RESIDE OTS^30^ to train our dust removal network. Then, we synthesized the sand dust images based on RESIDE^30^ to evaluate the performance of the proposed fusion algorithms.

### Implementation details

In the experiments, all the parameters in the network are initialized with Gaussian random variables; For training the network, the images are cropped to $128\times 128\times 3$; The batch size is set to 16; To lighten the network, we mapped the dimension of latent vector in representation space to 32; Adam optimizer with an initial learning rate of 0.001 is used to update the parameters. The experiments were conducted on a PC with an Intel(R) Core (TM) i5-9400 CPU@2.90GHz, 16GB RAM, and network on an NVIDIA GeForce RTX2070 GPU.

### Sand dust removal on synthetic dataset

Due to the specific nature of the sand dust images, there is no publicly available dataset for evaluating the algorithms. So, we synthesized the sand dust images based on RESIDE SOTS^30^ to calculate the full-reference metrics. Considering the characteristics of low brightness and contrast of dust degraded image, we randomly select $\alpha _1$, $\alpha _2$ and $\alpha _3$ from the uniform distribution interval [0.85, 0.9], [0.6, 0.65], [0.25, 0.3] as the attenuation coefficients of R, G, B channels respectively to make the synthesized images more realistic in vision.

**Figure 7:**
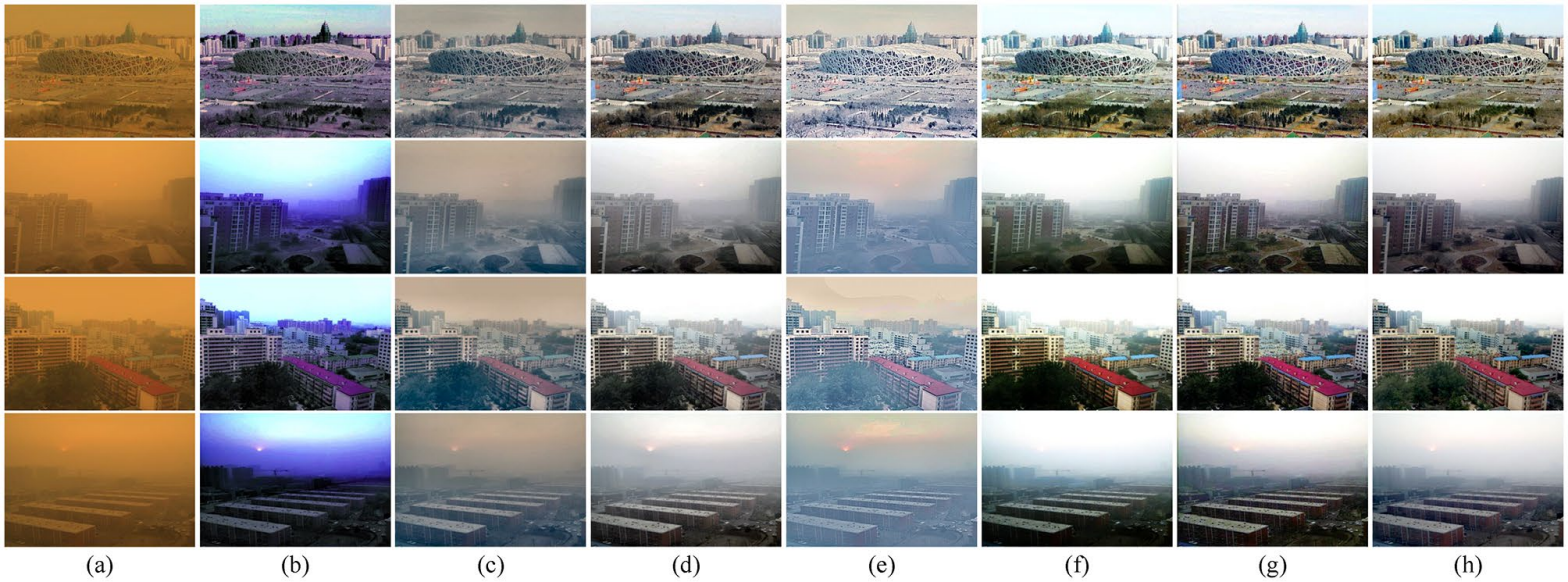
The comparisons on synthetic sand dust images. (**a**) The sand dust images. (**b**) CIDC^16^. (**c**) NGT^19^. (**d**) FBE^34^. (**e**) HRDCP^15^. (**f**) TTFIO^23^. (**g**) The proposed method. (**h**) The reference images.

We compared the proposed method with the prior sand dust removal algorithms including CIDC^16^, NGT^19^, FBE^34^, HRDCP^15^ and TTFIO^23^. For the fairness of comparison, all the code of the comparison algorithms is from the website of their authors. The comparative results of the qualitative analysis on the synthetic dataset are shown in Fig. 7. One can see that CIDC^16^ cannot eliminate the influence of color deviation, it over-enhanced the blue channel component, resulting in an overall blue-violet tint to the images and the results still have obvious color distortion. Furthermore, it seems that the results of NGT^19^ and HRDCP^15^ are grayed out, the contrast of the processed images is too low and the details are not prominent, there are no significant visual improvements in both of their results. FBE^34^ can solve the color distortion issue very well, and the images are visually improved. Nevertheless, the algorithm only removes the sand, there is still a certain sense of haziness in vision. TTFIO^23^ can correct the color of the degraded image and remove dust to some extent, but the outputs appear visually unnatural due to the brighter sky area. The comprehensive comparison shows that the proposed method can effectively improve the clarity of the sand dust images. The residual structure of the network plays a positive role in balancing the brightness and the contrast of the images, making the outputs closer to the reference images.

To quantitatively evaluate the performance of the comparison algorithms, we adopted Peak Signal to Noise Ratio (PSNR), Structural Dissimilarity (DSSIM), CIEDE2000, and CIE94 as the metrics. PSNR and DSSIM are widely used to measure the image quality, while CIEDE2000 and CIE94 are commonly adopted to evaluate the image tones. The higher metrics of PSNR and the lower DSSIM, CIEDE2000, and CIE94 denote the better performance of the algorithm. The comparison metrics are listed in Table 1. One can see that compared with the prior algorithms, our method achieved the best performance in PSNR, CIEDE2000, and CIE94. The quantitative evaluation experiments show the outstanding performance of the proposed method.

**Table 1:** Quantitative comparisons with the prior methods on synthetic dataset.

Methods	PSNR(↑)	DSSIM(↓)	CIEDE2000(↓)	CIE94(↓)
CIDC^16^	15.8217	0.2617	42.3948	22.3435
NGT^19^	15.7442	0.2525	30.4524	17.0162
FBE^34^	$\underline{20.7207}$	**0.1047**	$\underline{23.9693}$	$\underline{10.9815}$
HRDCP^15^	12.9239	0.291	36.7435	23.6501
TTFIO^23^	20.115	0.1182	30.509	11.2424
Proposed method	**20.8125**	$\underline{0.1125}$	**23.77**	**10.5483**

Bold values indicate the best performance and the underlined values indicate the second best.

### Sand dust removal on real-world degraded image

**Figure 8:**
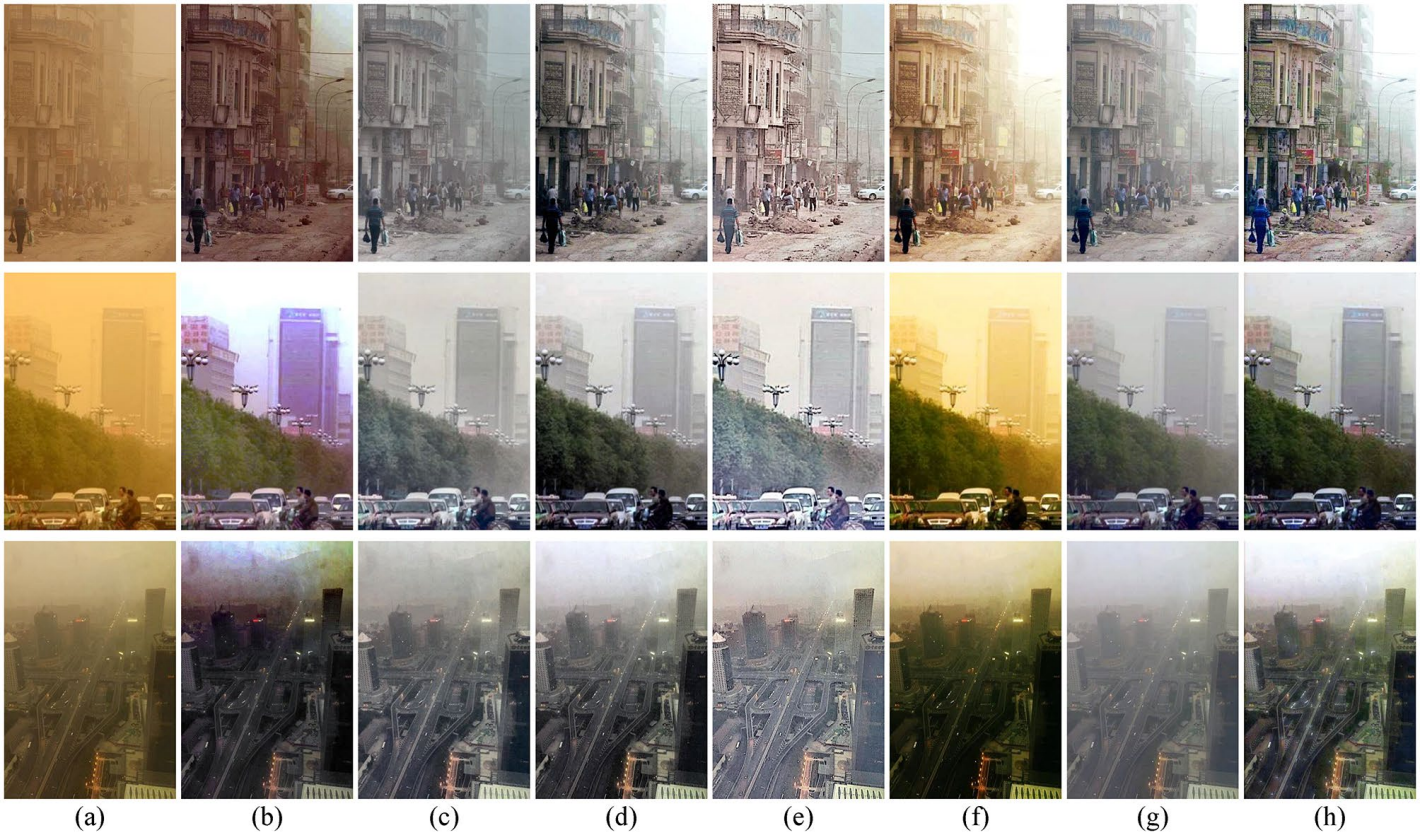
The comparisons of sand dust removal on real-world images. (**a**) Real-world sand dust images. (**b**) CIDC^16^. (**c**) NGT^19^. (**d**) FBE^34^. (**e**) HRDCP^15^. (**f**) TTFIO^23^. (**g**) Only color correction. (**h**) The proposed method.

**Figure 9:**
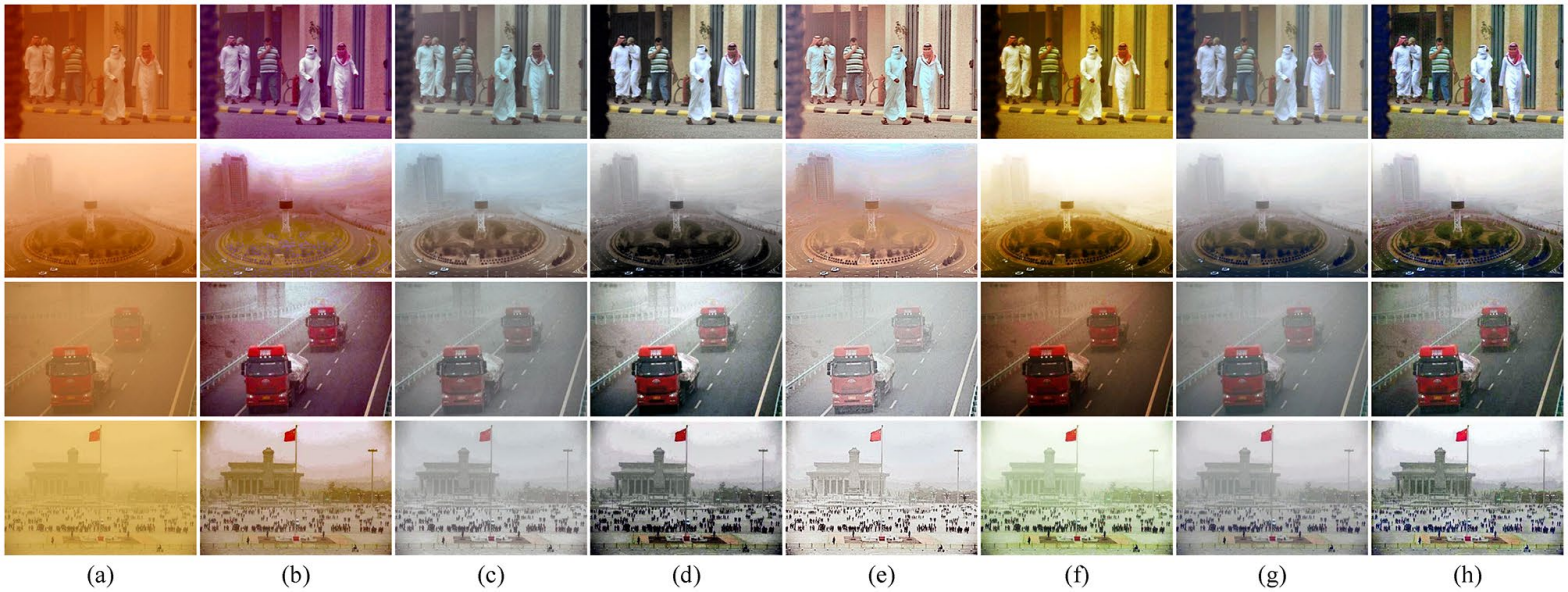
The comparisons of sand dust removal on real-world images. (**a**) Real-world sand dust images. (**b**) CIDC^16^. (**c**) NGT^19^. (**d**) FBE^34^. (**e**) HRDCP^15^. (**f**) TTFIO^23^. (**g**) Only color correction. (**h**) The proposed method.

In sandstorm weather, the concentration of sand dust images is not evenly distributed, which leads to a larger color deviation range of the real-world degraded images than the synthetic images. The difficulty of sand dust removal in real scene images is significantly higher than in synthetic images. To further evaluate the performance of the algorithms, it is necessary to conduct comparisons on real-world sand dust images. We provide several comparative examples of real-world degraded images with different visibility levels, as shown in Fig. 8. CIDC^16^ and TTFIO^23^ can remove the dust and improve the clarity of the image, but the images processed by the algorithms still have obvious color distortion. Although NGT^19^ and HRDCP^15^ can balance the hue and brightness of the images the contrast of the results is too low and the results are still hazy in vision. The outputs of FBE^34^ are better than other comparison algorithms, however, the brightness in the local area is too dark. Visually, the proposed color correction algorithm can effectively correct the tone of the images. By combining the correction algorithm with CNN, the clarity of the degraded images can be further improved. Please zoom in for a better illustration, the proposed algorithm can remove the haze more thoroughly and generate more natural results with less color distortion. As shown in Fig. 9, we provide some comparative examples under different sandstorm weather to further demonstrate the generalization capabilities of the proposed method. In Fig. 9, the proposed fusion algorithm is superior in terms of image detail preservation and color fidelity.

We adopted the non-reference evaluation metrics including Natural Image Quality Evaluator (NIQE)^35^, Spatial-Spectral Entropy-based Quality (SSEQ)^36^ and Blind Image Quality Indices (BIQI)^37^ to objectively evaluate the performance of the algorithm on real-world sand dust images. As shown in Table 2, the smaller NIQE, SSEQ, and BIQI metrics represent the better performance of the algorithm. In Table 2, one can see that compared with the prior methods, the proposed fusion algorithm still performs well even in the more complex real-world degraded sand dust images.

**Table 2:** The non-reference evaluation metrics on real-world sand dust images.

Methods	NIQE(↓)	SSEQ(↓)	BIQI(↓)
CIDC^16^	3.3061	$\underline{30.476}$	28.5029
NGT^19^	3.4346	34.2992	28.8722
FBE^34^	$\underline{3.258}$	31.2077	$\underline{27.7053}$
HRDCP^15^	3.5265	32.3583	32.0626
TTFIO^23^	3.5427	33.6551	28.9912
Proposed method	**3.0908**	**30.3133**	**26.0908**

Bold values indicate the best performance and the underlined values indicate the second best.

## Ablation study

There is a doubt that why do we need to first correct the color distorted channel and then improve the visibility using CNN rather than direct optimization for sand dust image with CNN? In this section, we will further analyze and discuss the effect of the fusion strategy. Specifically, there are three different ways: (1) Color correction; (2) End-to-end CNN; (3) Fusion strategy (Proposed method). We synthesized the training dataset for the training network using the method mentioned in “Sand dust removal on synthetic dataset” section, and compare (2) with (3) at the same training iterations.

**Figure 10:**
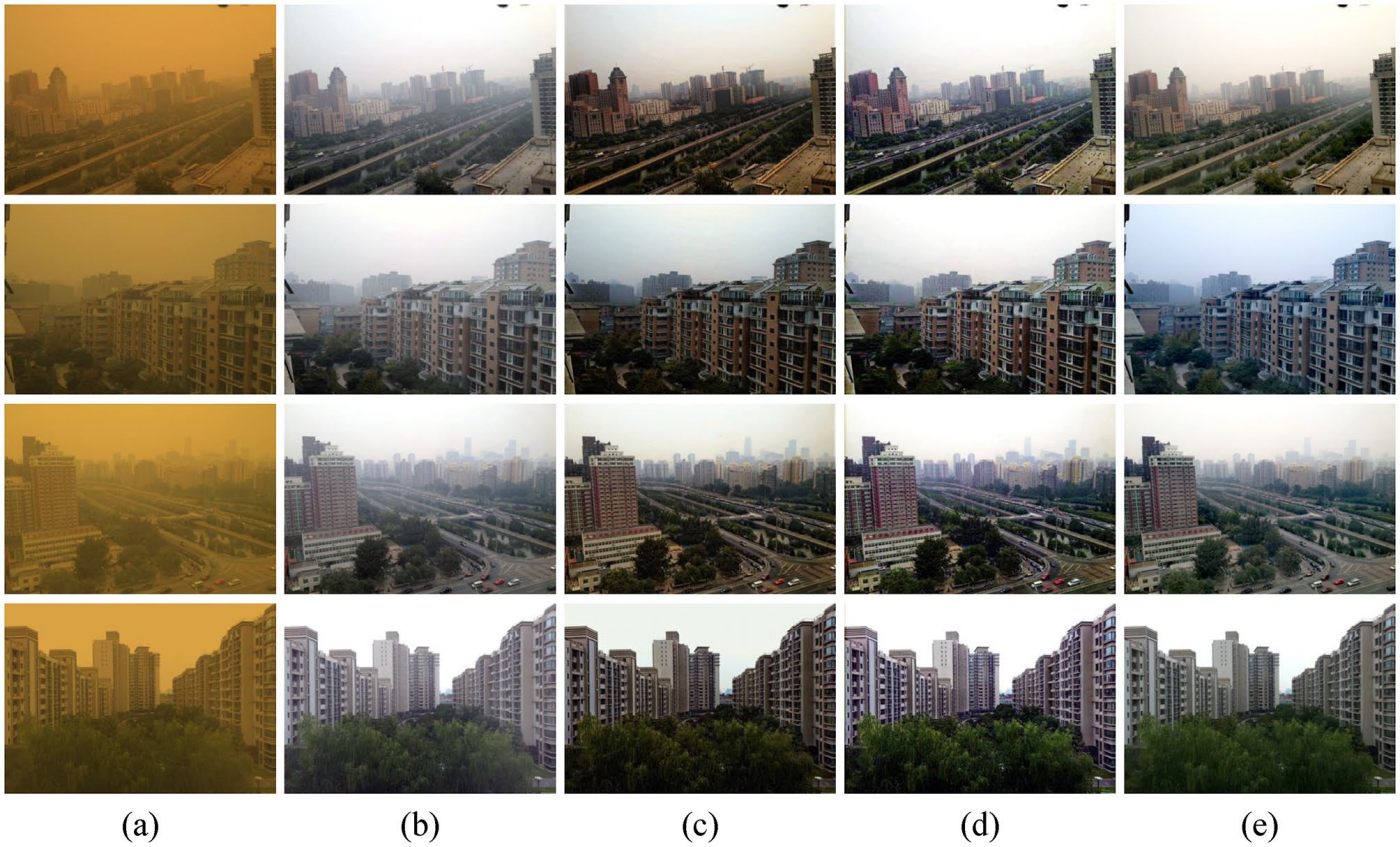
Comparisons of ablation study using synthetic sand dust images. (**a**) Sand dust images. (**b**) Color correction. (**c**) End-to-end CNN. (**d**) Fusion strategy. (**e**) The reference images.

The qualitative comparisons for the ablation study on the synthetic dataset are shown in Fig. 10. The color correction algorithm can balance the hue of sand, but it cannot remove the dust thoroughly. For end-to-end CNN, it can eliminate the effects of sand dust, providing a significant visual improvement. But the results of end-to-end CNN are too dark and there exists slight color shifting compared to the reference image. The fusion strategy can effectively solve the above issues and the results are visually closer to the reference image. Table 3 shows the objective evaluation indicators.

**Figure 11:**
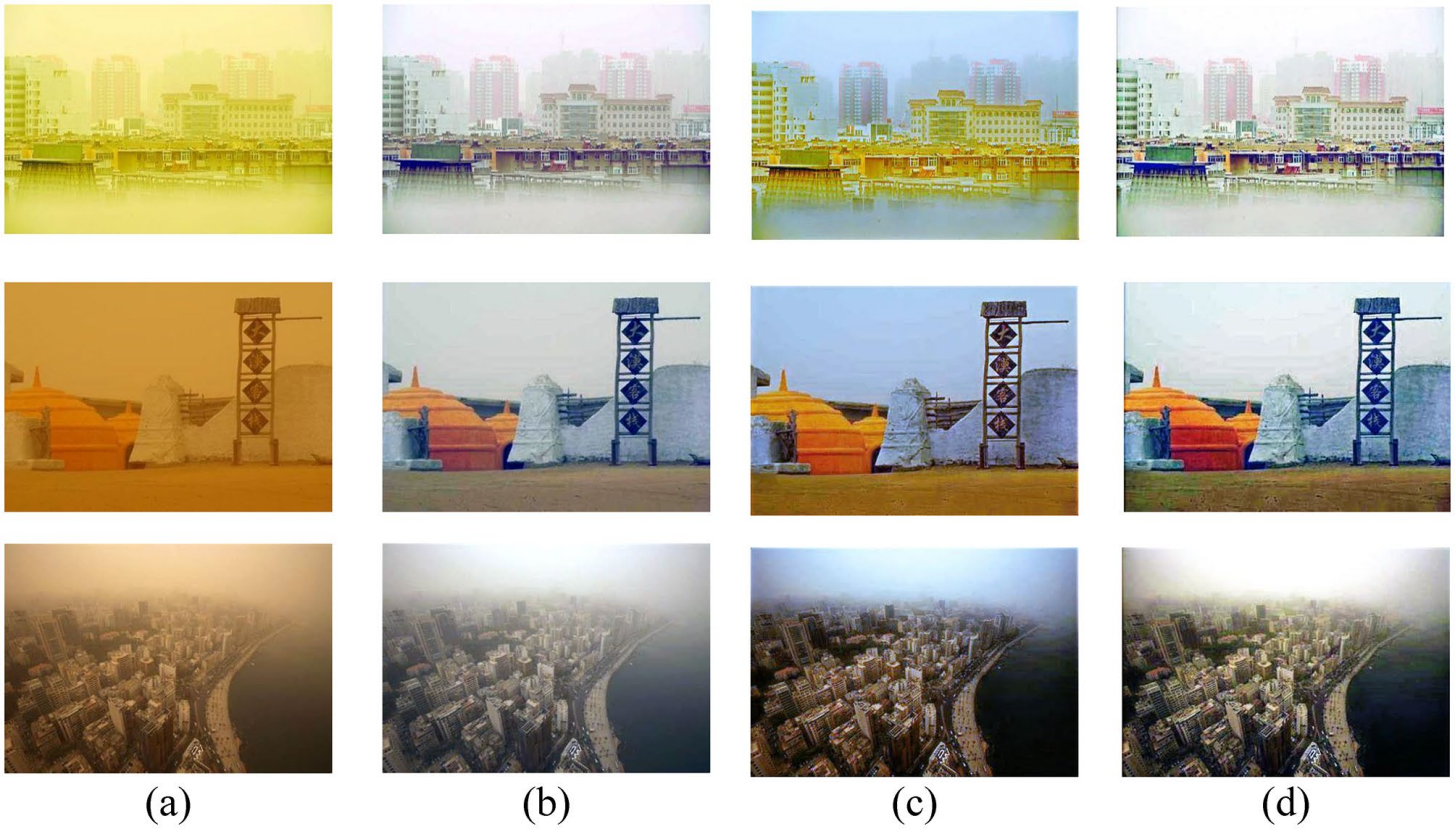
Comparisons of ablation study using real-world sand dust images. (**a**) Sand dust images. (**b**) Color correction. (**c**) End-to-end CNN. (**d**) Fusion strategy.

In addition, we also made comparisons on real-world sand dust images. As shown in Fig. 11, one can see that the color correction algorithm can remove the sand. However, the processed images are still hazy in vision. From the first row, there is significant color distortion in the result of end-to-end CNN. In the sky area of the middle and bottom rows, the results of end-to-end CNN still exist with residual color distortion. Compared with the color correction algorithm and end-to-end CNN, the fusion strategy can effectively remove the sand dust, while better restoring the original hue and texture details of the images.

**Table 3:** Objective evaluation indicators of ablation study using synthesized sand dust images.

Methods	PSNR(↑)	DSSIM(↓)	CIEDE2000(↓)	CIE94(↓)
Color correction	$\underline{20.5946}$	0.1242	26.4625	$\underline{11.0981}$
End-to-end CNN	20.5207	$\underline{0.1155}$	$\underline{25.6695}$	11.2113
Fusion strategy	**20.8125**	**0.1125**	**23.77**	**10.5483**

Bold values indicate the best performance and the underlined values indicate the second best.

For the above experimental results, we conclude that, on the one hand, the proposed end-to-end CNN may not be compatible with this sand dust removal task. On the other hand, as we all know, CNN has a strong ability to fit the training data, but the generalization ability of CNN is often limited by the dataset. Even though we synthesized the sand dust images visually close to the real image, the synthetic dataset is not representative of all real-world sandstorm scenes due to the complex distribution of sand dust.

## Conclusion

In this paper, we proposed a novel fusion strategy for single image sand dust removal task. The proposed method can effectively solve the issues of color distortion and local information loss widely existing in the prior sand dust removal algorithms. The residual network can suppress the noise, adaptively adjust the brightness and stretch the contrast of the images. The comprehensive experiments show that, compared with the prior algorithms, the proposed method can effectively improve the clarity of sand dust degraded image. The local details of the images are more prominent, and the overall tone is more in line with the visual characteristics of human eyes. However, the implementation of end-to-end neural networks is remain hampered by the fact that there is no publicly available sandstorm dataset, and we are still exploring a more theoretical synthesis method to promote future research on sand dust image enhancement. Based on our evaluation and analysis, several overarching observations and empirical findings are summarized as:

- The imaging mechanism of sand dust images should be deeply studied based on atmospheric scattering models, and the distribution of more sand dust degradation scenarios should be taken into account.
- The traditional algorithm is used to preprocess the image, and the CNN is employed to process the complex nonlinear mapping, which can improve the robustness of the algorithm and reduce the learning pressure of the neural network. This fusion strategy provides a novel way to solve such challenging image enhancement tasks.
- The appropriate prior theory may play a positive role in training CNN. We advocate a combination of appropriate priors and learning algorithms to make their advantages complementary.

## Data Availability

The datasets used and/or analysed during the current study available from the corresponding author on reasonable request.
